# Development and validation of the Ulcerative Colitis patient-reported outcomes signs and symptoms (UC-pro/SS) diary

**DOI:** 10.1186/s41687-018-0049-2

**Published:** 2018-05-30

**Authors:** Peter D. R. Higgins, Gale Harding, Dennis A. Revicki, Gary Globe, Donald L. Patrick, Kristina Fitzgerald, Hema Viswanathan, Sarah M. Donelson, Brian G. Ortmeier, Wen Hung Chen, Nancy K. Leidy, Kendra DeBusk

**Affiliations:** 1 University of Michigan, Ann Arbor, MI USA; 2 Evidera, Bethesda, MD USA; 3 Amgen Inc., Thousand Oaks, CA USA; 4 University of Washington, Seattle, WA USA; 5 Genentech Inc., South San Francisco, CA USA; 6 Present address: Allergan Inc, Irvine, CA USA; 7 Present address: Office of New Drugs, CDER, Silver Spring, USA

**Keywords:** Ulcerative colitis, Patient-reported outcomes, Signs and symptoms, Reliability, Validity, Clinical trial endpoints

## Abstract

**Background:**

The clinical course of ulcerative colitis (UC) and the effects of treatment are assessed through patient-reported signs and symptoms (S&S), and endoscopic evidence of inflammation. The Ulcerative Colitis Patient-Reported Outcomes Signs and Symptoms (UC-PRO/SS) measure was developed to standardize the quantification of gastrointestinal S&S of UC in clinical trials through direct report from patient ratings.

**Design:**

The UC-PRO/SS was developed by collecting data from concept elicitation (focus groups, and individual interviews), then refined through a process of cognitive interviews of 57 UC patients. Measurement properties, including item-level statistics, scaling structure, reliability, and validity, were evaluated in an observational, four-week study of adults with mild to severe UC (*N* = 200).

**Results:**

Findings from qualitative focus groups and interviews identified nine symptom items covering bowel and abdominal symptoms. The final UC-PRO/SS daily diary includes two scales: Bowel S&S (six items) and Abdominal Symptoms (three items), each scored separately. Each scale showed evidence of adequate reliability (α = 80 and 0.66, respectively); reproducibility (intraclass correlation coefficient = 0.81, 0.71) and validity, including moderate-to-high correlations with the Partial Mayo Score (0.79; 0.45) and Inflammatory Bowel Disease Questionnaire (IBDQ) total score (− 0.70; − 0.61). Scores discriminated by level of disease severity, as defined by the Partial Mayo Score, Patient Global Rating, and Clinician Global Rating (*p* < 0.0001).

**Conclusions:**

Results suggest that the UC-PRO/SS is a reliable and valid measure of gastrointestinal symptom severity in UC patients. Additional longitudinal data are needed to evaluate the ability of the UC-PRO/SS scores to detect responsiveness and inform the selection of responder definitions.

**Electronic supplementary material:**

The online version of this article (10.1186/s41687-018-0049-2) contains supplementary material, which is available to authorized users.

## Significance of this study

What is already known about this subject?

- The US Food and Drug Administration (FDA) has established a pathway for rigorous development of disease-specific Patient-reported Outcome (PRO) tools for clinical trials and clinical use.
- Currently, there are no measures developed and validated according to the FDA PRO guidance available to assess the symptoms of ulcerative colitis (UC).

What are the new findings?

- Using the US FDA pathway for rigorous development of disease-specific PRO tools, we have developed and validated a new patient-reported sign and symptoms measure for clinical trials and clinical use in UC.
- This is the first symptom measure of UC to meet US FDA PRO guidelines.
- This modular instrument can be used with appropriate individual modules customized to the mechanism of action of a candidate therapy, from purely anti-inflammatory medications, to those targeting pain, dysmotility, or functional symptoms.

How might it impact on clinical practice in the foreseeable future?

- Using electronic device systems, PROs in IBD can be routinely measured before and between appointments in order to identify response to therapies or failure of therapies.

## Background

Ulcerative colitis (UC) is a chronic, relapsing inflammatory disease of the colonic mucosa [1]. Recent studies estimate that 700,000 people are afflicted in the United States (US) and Canada alone [2], with a global annual incidence ranging from 0.5 to 24.5 cases per 100,000 [3]. The characteristic signs and symptoms of UC include abdominal pain, frequent diarrhea, urgent bowel movements, and rectal bleeding, which are not only disconcerting to patients, but can adversely affect their quality of life [4].

Clinically, UC is monitored through signs and symptoms of disease activity and periodic objective assessment (e.g., an endoscopy) to evaluate mucosal inflammation. In clinical trial settings, the Mayo Score historically has been used to assess disease activity, combining endoscopic findings with physician-rated signs and symptoms, based on information provided by the patient in a single total score.

In 2009, the US Food and Drug Administration (FDA) released a guidance for the development of patient-reported outcome (PRO) measures to support labeling claims for new medical treatments and products [5]. This guidance emphasizes the importance of conducting qualitative research throughout the process of instrument development to ensure that the content of the measure is consistent with the patient experience and covers what they consider most important about a condition and/or treatment intervention. Quantitative work to assess the instrument’s psychometric properties, such as reliability and validity, is also recommended. This standard in instrument development is an increasing regulatory requirement for efficacy evaluation and labeling purposes for treatment interventions [5, 6]. Composite measures that combine different aspects of a disease, such as clinically derived signs, patient symptoms, and/or clinical tests, are now viewed by the FDA as concepts that are best measured, scored, and reported separately. Furthermore, both the FDA and European Medicines Agency have recently released guidelines specific to clinical trials of ulcerative colitis, noting the importance of including an adequately validated PRO to assess symptomatic relief as a primary outcome measure in pivotal clinical trials of UC [7, 8]. For these reasons, a new patient-reported sign and symptom measure for UC was developed and validated according to the US FDA PRO Guidance and is the first symptom measure of UC to meet these guidelines.

The Ulcerative Colitis Patient-reported Outcomes (UC-PRO) instrument was designed to comprehensively assess the signs, symptoms, and impact of UC through six modules. Modules 1 (Bowel Signs and Symptoms) and 2 (Abdominal Symptoms) comprise the UC-PRO Signs and Symptoms (UC-PRO/SS) measure. Module 3 addresses Systemic Symptoms, Module 4 addresses Coping Strategies, Module 5 addresses Daily Life Impact, and Module 6 covers Emotional Impact. Any or all of these modules may be used in any given study.

The focus of this paper is on evaluating the UC-PRO/SS measure in terms of treatment-related outcomes and supporting potential labeling claims related to the gastrointestinal (GI) signs of symptoms of UC from the perspective of the patient. The UC-PRO/SS was developed to quantify the signs and symptoms in clinical trials of adults (18 years of age or older) with moderate-to-severe UC treated in outpatient settings. This paper describes the development and initial validation of this instrument. Given the variability in the symptomatic experience of this patient population, the UC-PRO/SS is completed as a daily diary, and is designed for electronic administration.

As noted throughout the paper, details related to the UC-PRO/SS development and validation are provided in the online Supplementary Material (Additional file 1). Also included in the Supplementary Material (Additional file 1) is information on the Systemic Symptoms scale (Module 3 of the UC-PRO), a five item scale that can be included as part of the daily diary to evaluate the non-gastrointestinal systemic symptoms of UC. Based on the qualitative work, these symptoms were found to be relevant and important to the patient experience. However, systemic symptoms are generally not affected by current gut-specific agents. From a regulatory perspective, such symptoms are considered “distal” to the target disease activity and are therefore less suitable for testing treatment effects and/or inclusion in a product label. Because the intent is to use the UC-PRO/SS in drug development trials, with the qualification of the instrument as a Drug Development Tool for this purpose currently underway [9], Module 3, Systemic Symptoms, is not included in the CD-PRO/SS measures. At the discretion of the user/sponsor, it can be administered as part of a diary and serve as an exploratory assessment in clinical trials. This scale may also be useful in studies or clinical trials evaluating the systemic component of UC. Information in the online Supplementary Material (Additional file 1) is intended to facilitate use of this Module.

## Methods

The research was conducted in two phases, consistent with the methodology outlined by the FDA PRO Guidance [5]. Phase I addressed the content and structure of the measure, and the documentation of content validity through qualitative research methods. Phase II was a four-week observational study to address its measurement properties, including scoring and evaluation of reliability and validity. All data collection and recruitment procedures met institutional review board (IRB) and Health Insurance Portability and Accountability Act requirements, and all applicable state and federal laws and regulations. Study protocols were approved by an independent IRB and written informed consent was obtained from study subjects prior to completing any study related activities.

For each phase, subjects were recruited from US gastroenterology clinics and included ambulatory adult patients with clinician-confirmed UC, based on available biopsy. Patients participating in an interventional study were excluded, as were those with an ileostomy, colostomy, or who had an intra-abdominal surgery in the 4 months prior to screening. Patients represented a range of disease activity, from mild to severe, based on the Simple Clinical Colitis Activity Index (SCCAI) or Partial Mayo score [10].

### Phase I: Qualitative – Development and content validity

A two-stage qualitative research process was used to determine instrument content, and to ensure clarity and understanding in the target patient population. Focus groups and interviews were conducted by experienced study team members using a semi-structured discussion guide, informed by clinical expert input and a review of the literature to cross reference symptoms, and were audio-recorded and transcribed for analysis. Additionally, participants completed a sociodemographic questionnaire for use in characterizing the study sample. Additional methods are outlined below, with details provided in the online Supplementary Material (Additional file 1).

#### Stage 1: Focus groups and one-to-one interviews

Six focus groups (*n* = 33) and one-to-one qualitative interviews (*n* = 9) were conducted to identify important UC symptoms, explore the frequency and variability of these symptoms, and inform the development of response options and appropriate recall for a symptom measure in the target population. Subjects were recruited from seven US gastroenterology sites to capture diversity in terms of race, ethnicity, and geographic location. In addition, subjects represented a range of disease activity, based on the SCCAI for focus group participants (SCCAI ≤5, n = 3; SCCAI 6–8, *n* = 5; and SCCAI ≥8, *n* = 23 [SCCAI data were missing for two participants]) and the Partial Mayo Scores for those participating in one-to-one interviews (Partial Mayo Score 2–4, n = 5; Partial Mayo Score ≥ 4, *n* = 4). Discussion focused on participants’ current symptom experiences, their experiences during an episode or flare-up, and the impact of these symptoms on their daily life.

Content analyses were performed by independent coders, with data organized using qualitative software (NVivo or ATLAS.ti). At each stage of instrument development, participant quotes were grouped and summarized by thematic code to assess the saturation of concepts. Saturation is defined as the point at which no substantially new themes, descriptions of a concept, or terms are introduced as additional discussions are conducted [11].

Results were discussed with clinical experts and used to generate a list of relevant symptoms and a draft UC-PRO/SS measure, including instructions, items, and response options.

#### Stage 2: Cognitive interviews

Two rounds of cognitive interviews (*n* = 15) were conducted at three US clinical sites to examine the relevance, comprehensiveness, and clarity of the draft UC-PRO/SS (including systemic symptoms), and to refine the measure as needed. Subjects were asked to complete the questionnaire independently and were then interviewed about the content, including instructions, recall period, candidate items, and response options. Upon completion of 10 interviews (Round 1), the instrument was edited for clarity based on subject comments, and the revised instrument was evaluated by a new sample of UC patients (Round 2, *n* = 5). Round 2 also provided an opportunity to examine patient understanding of the scales formatted as ePRO screen shots for use as an electronic daily diary, with one item per page. Upon completion of this set of interviews, the instrument was finalized for psychometric evaluation.

### Phase II: Quantitative – Reliability and validity

An observational, prospective, four-week study was conducted to examine the reliability and validity of the UC-PRO/SS in ambulatory adults with clinician-confirmed UC based on a biopsy obtained at least 3 months prior to study screening. Participants represented a range of disease severity based on the Partial Mayo Score (0–2, *n* = 56 [28%]; 3–5, *n* = 88 [44%]; ≥6, n = 56 [28%]). Subjects were recruited for the psychometric study (Phase II) from 22 study sites in diverse regions of the US. Each participated in three protocol-driven clinic visits: Day 1 (Enrollment: Visit 1), Day 7 ± 3 days (Visit 2), and Day 28 ± 4 days (Visit 3).

#### Measures

Subjects completed the UC-PRO/SS (9 candidate items) and Module 3 Systemic Symptoms (5 candidate items), a patient global rating of disease severity, and a single item to assess the “worst pain” [12] each day during the 30-day study period, using an electronic hand-held device given to the subject upon enrollment; training was provided by clinical site personnel. In addition, subjects completed the paper-pen Partial Mayo Symptom Diary 7 days prior to Visits 2 and 3.

For score validation purposes, and to coincide with the clinician assessment, the following paper-pen questionnaires were completed by subjects at Visits 2 and 3, prior to seeing the clinician: Inflammatory Bowel Disease Questionnaire – 32 Items (IBDQ-32) [13, 14], Work Productivity and Activity Impairment – Specific Health Problem (WPAI-SHP) [15], Patient-Reported Outcomes Measurement Information System (PROMIS) Global Health Scale [16], a patient global rating of disease severity, and a patient global rate of change in disease severity.

Clinicians completed the Partial Mayo Score at Visits 2 and 3 based on their assessment of patients’ answers to the paper-pen Partial Mayo Symptom Diary and a clinical assessment after seeing the patient. This measure is highly correlated (0.71) with the full Mayo score [10], which includes flexible sigmoidoscopy. In addition, clinicians completed a clinician global rating of disease severity at each clinic visit, and a clinician global rating of changes in disease severity at clinic Visits 2 and 3.

### Statistical analysis

Analyses were performed in accordance with a pre-specified statistical analysis plan. SAS version 9.2 was used for all statistical analyses, excepting the confirmatory factor analysis (conducted with Mplus) [17], and the Rasch analysis (conducted with RUMM2030) [18]. Item-level analyses were evaluated using the “worst” day between (and inclusive of) Visit 1 and Visit 2, defined as the day with the worst rating on the patient global rating of disease severity. These analyses included measures of central tendency, floor and ceiling effects, and inter-item correlations. An item was flagged for potential problems if it showed a floor (minimum response > 25%) or ceiling effect (maximum response > 25%), or when the inter-item correlation was greater than 0.80. Confirmatory and exploratory factor analyses were performed to evaluate the structure of the measure and develop a scoring algorithm. Rasch analyses were conducted separately for each factor that consisted of a single dimension; items with negative fit residual value ≤ − 3.0 or ≥ 3.0 positive fit residual were flagged for potential deletion [19].

After the items and scales were finalized, scores were evaluated for reliability and validity. Specifically, internal consistency reliability was assessed using Cronbach’s alpha coefficient, with a target value of 0.7 indicating good internal consistency [20, 21]. Test-retest reliability was assessed between Day 1 and Day 7 among those with no change in patient-rated global rating of change in UC severity at Visit 2. Intraclass correlation coefficients (ICC) were computed, where ≥0.7 indicates adequate reproducibility [21, 22].

Score validity was assessed by examining correlations of the UC-PRO/SS with the Partial Mayo Score; IBDQ; WPAI-SHP; PROMIS measures of global physical health (GPH), global mental health (GMH), general health, and satisfaction with social role scores; worst pain; and patient and clinician global ratings of disease severity. The UC-PRO/SS was expected to be moderately-to-highly correlated (> 0.30) with Partial Mayo Scores and moderately correlated (0.30–0.50) with IBDQ scores, worst pain, PROMIS GPH, and patient-rated global ratings of disease severity, demonstrating convergent validity [23]. Lower correlations were anticipated between the UC-PRO/SS scales and PROMIS GMH and satisfaction scores, and WPAI-SHP scores (overall work impairment and activity impairment) (≤0.30), as these concepts were thought to be more distal to the symptom experience.

Known-groups validity was examined to determine whether the UC-PRO/SS could distinguish between patients by disease severity, defined in three ways: 1) by Partial Mayo Scores (mild, score 0–4; moderate, score 5–7, severe, score ≥ 8); 2) by clinician-rated global assessment of disease severity; and 3) by patient-rated disease severity. Analysis of covariance models with baseline clinical measurement group as the main effects in the model were used, adjusting for age and gender.

## Results

### Study samples

Demographics and clinical characteristics for the study samples by phase are shown in Table 1. The study subjects ranged in age from 21 to 80 years of age, representing a range of ethnicity, race, extent of disease, and disease severity (at baseline).

**Table 1:** Patient Demographic and Clinical Characteristics, by Study Phase*

Characteristics	Phase I: Qualitative Development and Content Validity (*n* = 57)	Phase II: Quantitative Score Reliability and Validity (*n* = 200)
Age in years, Mean (SD) [Range]	44.1 (13.8) [21–77]	45.7 (14.60) [21–80]
Gender, n (%)		
Female	36 (63%)	117 (59%)
Ethnicity, n (%)		
Hispanic or Latino	6 (10%)	42 (21%)
Not Hispanic or Latino	50 (88%)	155 (78%)
Missing	1 (2%)	3 (2%)
Race, n (%)^†^		
American Indian or Alaska Native	1 (2%)	2 (10%)
Asian	1 (2%)	11 (6%)
Black or African American	3 (5%)	28 (14%)
Native Hawaiian or other Pacific Islander	1 (2%)	2 (1%)
White	43 (75%)	153 (77%)
Other	6 (10%)	6 (3%)
Missing	2 (4%)	0 (0%)
Extent of Disease, n (%)^a^		
Ulcerative proctitis	4 (7%)	19 (10%)
Proctosigmoiditis	9 (16%)	62 (31%)
Left-sided colitis	14 (25%)	59 (30%)
Extensive colitis	2 (4%)	18 (9%)
Pancolitis	25 (44%)	42 (21%)
Missing or unknown	3 (5%)	0 (0%)

*Abbreviations*: *n* number, *SD* standard deviation

^a^Percents do not add to 100 due to rounding

^b^Subjects able to choose more than one race

#### Phase I: Development and content validity

Findings from focus groups and individual interviews identified nine sign and symptom items covering bowel and abdominal symptoms. Important bowel-related symptoms from the perspective of the patient included frequency of bowel movements (BMs), consistency of BMs, the presence of blood, the presence of mucus, the urge/need to have a BM right away, and leakage/accidents. Key abdominal symptoms included pain in stomach area, bloating, and gas. The symptoms that were most relevant during flare-ups included blood in BMs, frequency of BMs, consistency of BMs, and urge/need to have a BM right away. Patient descriptions of the symptoms they experienced during a flare were similar to language they used to describe their everyday symptoms, just more severe and/or persistent. Patient descriptions of their symptom experience underline the variability not only within, but also between patients.

Additional details of the qualitative methods and results, along with evidence of saturation, are provided in the online Supplementary Material (Additional file 1).

The final version, which was ready for quantitative testing, was a daily diary that comprised nine candidate symptom items covering all GI signs and symptoms identified by patients and confirmed by clinicians as relevant and important to the assessment of disease activity in UC. For number and consistency of bowel movements, response options were based on frequency. The number of bowel movements was queried on a 8-point scale with ranges considered reasonable and meaningful to patients and clinicians (0, 1–2, 3–4, 5–6, 7–9, 10–12, 13–17, 18–24, more than 24). The intent was to use quantitative data to evaluate these categories, with the possibility of combining and/or deleting categories, while maintaining a clinically meaningful and sensitive indicator of bowel movement frequency. For all other symptoms, response options were based on presence (yes/no) and severity or frequency of each, with scores ranging from 0 (none or not at all) to 4 (always or very severe).

#### Phase II: Reliability and validity

##### Item and factor analysis and scoring algorithm

Item-by-item descriptive statistics are shown in Table 2. Subjects used the full range of response options for each item, with the exception of the item concerning number of bowel movements; as anticipated, no study participants reported zero BMs on their “worst” day between Visits 1 and 2. Six of nine items had a floor effect exceeding 25%, with nearly half of these outpatients reporting no leakage (62%), and no mucus (53%) or blood (47%) in their stool between Visit 1 and Visit 2 on their worst symptom day during this one-week observation period. There were no ceiling effects.

**Table 2:** Item Descriptive Characteristics at Worst Day between Visit 1 and Visit 2 (*N* = 198)

Item	Mean (SD)	Range	Floor (%)	Ceiling (%)	Missing (%)
Number of bowel movements	3.9 (1.60)	1–8	0 (0.0%)	6 (3.0%)	0 (0.0%)
Number of liquid bowel movements	1.8 (1.34)	0–4	48 (24.2%)	27 (13.6%)	0 (0.0%)
Blood in bowel movements	1.4 (1.48)	0–4	92 (46.5%)	23 (11.6%)	0 (0.0%)
Mucus in bowel movements	1.1 (1.36)	0–4	104 (52.5%)	14 (7.1%)	0 (0.0%)
Leak before reaching toilet	0.9 (1.21)	0–4	124 (62.6%)	6 (3.0%)	0 (0.0%)
Passing gas	2.2 (1.23)	0–4	29 (14.6%)	27 (13.6%)	0 (0.0%)
Need to have bowel movement right away	1.9 (1.35)	0–4	55 (27.8%)	20 (10.1%)	0 (0.0%)
Pain in belly	1.7 (1.25)	0–4	51 (25.8%)	14 (7.1%)	0 (0.0%)
Bloating in belly	1.5 (1.20)	0–4	60 (30.3%)	9 (4.5%)	0 (0.0%)

*Abbreviations*: *N* number, *SD* standard deviation

Findings support a two-factor solution, with confirmatory factor analyses subsequently conducted to determine goodness of fit statistics. One factor represents “Bowel Signs and Symptoms” and includes six items (Comparative Fit Index [CFI] = 0.98, Root Mean Square of Approximation [RMSEA] = 0.068, Weighted Root Mean Residual [WRMR] = 0.563), while the other factor represents “Abdominal Symptoms” and includes three items (CFI = 1.0, RMSEA = 0.0, WRMR = 0.0). Rasch analysis indicated that all of the fit residuals for items in each of the two models fell within the acceptable range (≥ − 3.0 and ≤ 3.0); however, several of the response categories were not ordered correctly, primarily due to very few responses for “rarely” and “mild” categories.

Taking into consideration findings from both the qualitative and quantitative studies, several decisions were reached regarding the UC-PRO/SS. First, given that few subjects (*n* = 6, 3%) endorsed the response category “more than 24” for the item “number of BMs,” this item response level was removed. Although a number of items demonstrated floor effects during the one-week observation, all were considered important from the perspective of the patient, based on qualitative studies, and clinically relevant. Finally, although the Rasch analyses suggested the number of response options for several items could be reduced from a 5- to a 4-point scale by combining responses, the distinction between “none” and “mild” and between “mild” and “moderate” was considered clinically important and the decision was made to retain the five-point scaling.

The final UC-PRO/GI-SS assesses two important indicators of disease activity in UC: Bowel Signs and Symptoms (six items) and Abdominal Symptoms (three items), each scored as a simple mean across all items comprising the scale. There is no single total score that combines both scales.

### Reliability

Adequate internal consistency was demonstrated with alpha coefficients of 0.80 for Bowel Signs and Symptoms and 0.66 for Abdominal Symptoms. Although findings indicate that the Cronbach’s alpha for the domain of Abdominal Symptoms would increase to 0.79 with the deletion of “passing gas,” the item was retained based on importance of this symptom from the patient perspective and expert opinion. Seven-day test-retest reliability in stable patients (*n* = 77 reporting no change in symptoms between Day 1 and Day 7) was supported with ICC values of 0.81 and 0.71 for Bowel Signs and Symptoms and Abdominal Symptoms, respectively.

### Validity

Correlations between the UC-PRO/SS domain scores and clinical and other relevant PRO measures are presented in Table 3. All relationships were confirmed based on a priori predictions, with both UC-PRO/SS scale scores demonstrating strong correlations with the Partial Mayo Score, IBDQ, worst pain, and patient and clinician global ratings of disease severity (convergent validity), and weaker correlations with measures of impact on daily life (discriminant validity).

**Table 3:** Correlations between UC-PRO/SS Scores and Other Clinical Variables^a,b^

Clinical Variable	Bowel Signs and Symptoms Rating (p value)	Abdominal Symptoms Rating (*p* value)
Clinician Ratings:		
Partial Mayo Score	0.79 (< 0.0001)	0.45 (< 0.0001)
Clinician Global Rating of Disease Severity	0.69 (< 0.0001)	0.44 (< 0.0001)
Patient Ratings:		
Patient Global Rating of Disease Severity	0.67 (< 0.0001)	0.52 (< 0.0001)
IBDQ		
Total score	−0.70 (< 0.0001)	− 0.61 (< 0.0001)
Bowel systems	− 0.73 (< 0.0001)	− 0.65 (< 0.0001)
Emotional health	− 0.61 (< 0.0001)	− 0.53 (< 0.0001)
Systemic systems	− 0.51 (< 0.0001)	−0.53 (< 0.0001)
Social function	−0.62 (< 0.0001)	−0.49 (< 0.0001)
WPAI-SHP		
Absenteeism	0.35 (< 0.0001)	0.22 (0.0107)
Presenteeism	0.63 (< 0.0001)	0.45 (< 0.0001)
Work productivity loss	0.63 (< 0.0001)	0.46 (< 0.0001)
Activity impairment	0.63 (< 0.0001)	0.47 (< 0.0001)
PROMIS		
Global physical health	−0.21 (0.0031)	− 0.28 (< 0.0001)
Global mental health	−0.28 (< 0.0001)	− 0.33 (< 0.0001)
General health	−0.26 (0.0003)	− 0.33 (< 0.0001)
Satisfaction with social role	− 0.25 (0.0006)	−0.28 (< 0.0001)
BPI–Worst Pain	0.57 (< 0.0001)	0.64 (< 0.0001)

*Abbreviations*: *BM* bowel movement, *BPI* brief pain inventory, *IBDQ* Inflammatory Bowel Disease Questionnaire, *PROMIS* Patient Reported Outcomes Measurement Information System, *UC-PRO/GI-SS* Ulcerative Colitis Patient-reported Outcomes Gastrointestinal Signs and Symptoms Scale, *WPAI-SHP* Work Productivity and Activity Impairment–Specific Health Problems

^a^Spearman’s correlation coefficients

^b^Seven-day average scores used

**Fig. 1:**
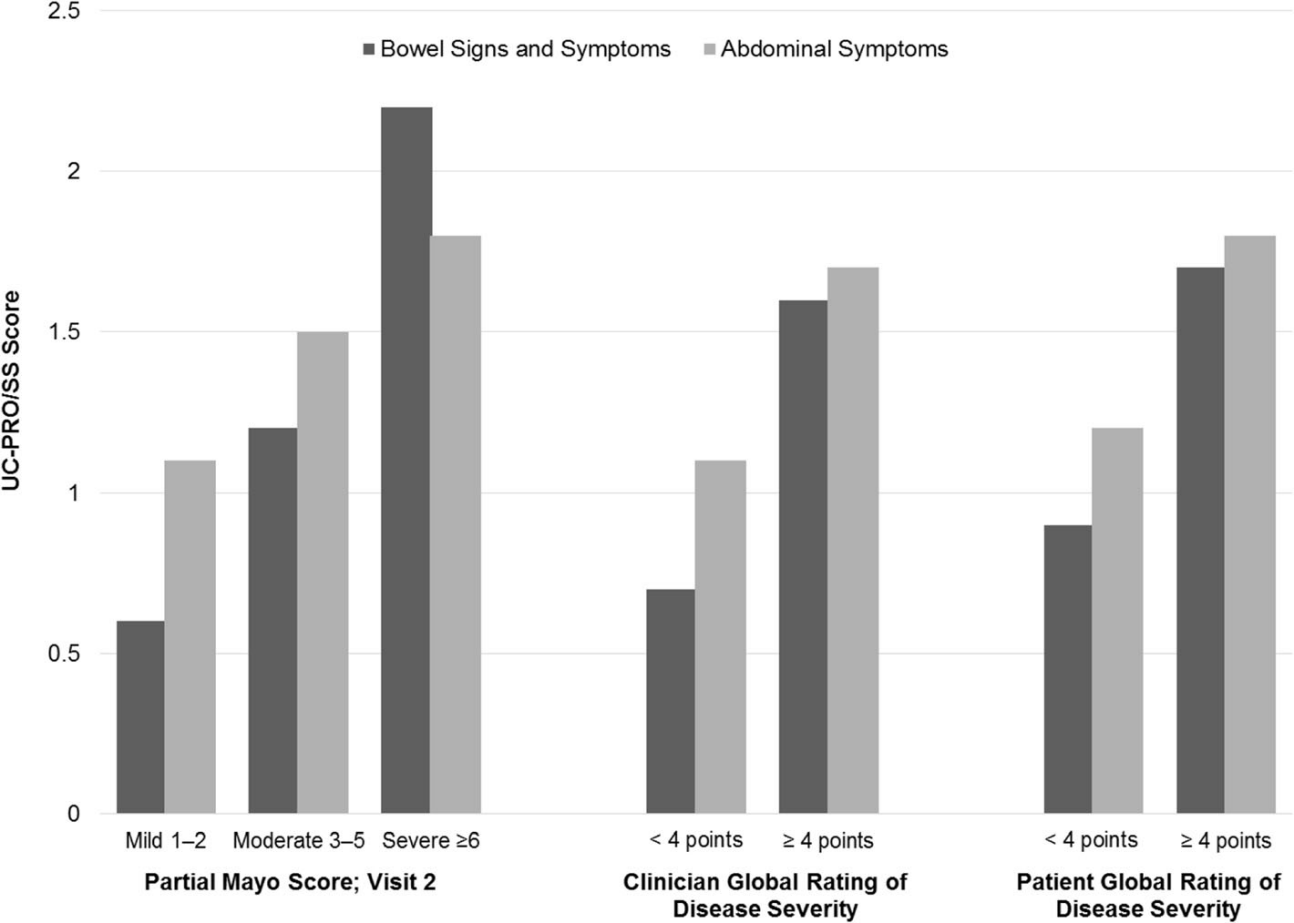
UC-PRO/SS Scores by Disease Activity

The Bowel Signs and Symptoms and the Abdominal Symptoms scales were each able to differentiate patients by symptom severity (*p* < 0.0001) based on the Partial Mayo Score, patient global rating of disease severity, and clinician global rating of disease severity (Fig. 1). Scale scores for both the UC-PRO/SS scales increased (indicating worse symptoms) with increasing Partial Mayo scores (indicating higher disease severity). Similarly, UC-PRO/SS scale scores were higher among patients who had patient global ratings of disease severity scores ≥ median compared to those with scores below the median. Similar findings were demonstrated based on clinician global rating of disease severity. Known-group validity tables are included in the online Supplementary Material (Additional file 1).

## Discussion

The UC-PRO/SS measure was developed to standardize the quantification of GI signs and symptoms of UC in clinical trials through direct patient ratings. The methodology used to develop the UC-PRO/SS followed the US FDA Guidance on PRO instrument development, which conveys the agency’s thinking on best practices for the development of measures and the evidence needed for the agency’s evaluation [5]. The UC-PRO/SS was developed based on data collection from concept elicitation and cognitive interviews of subjects with moderate to severe UC who were representative of the UC target population eligible for typical clinical trials. Measurement properties were tested in a four-week observational study of 200 adults with UC. The decision to retain or delete items for the final measure was an iterative process with consideration of floor and ceiling effects, results from the factor and Rasch analyses, previous qualitative results, and clinical considerations.

The psychometric evaluation study included patients with a history of very mild to moderately severe UC to capture responses from the full range of disease activity. The relatively large number of mild patients contributed to the floor effects observed across items, and the results of the Rasch analyses, which suggested little response distinction between “none” and “mild” or between “never” and “rarely.” Given the importance of these response categories from a clinical perspective and to capture degrees of improvement in more severe patients, these response options were retained, with the understanding that further evaluation will be needed to confirm their suitability and utility across populations with severely active disease.

The final UC-PRO/SS includes two scales: Bowel Signs and Symptoms (six items) and Abdominal Symptoms (three items), with both scales are scored separately. Performance testing of the UC-PRO/SS demonstrated evidence of internal consistency and reproducibility. The UC-PRO/SS scale scores showed moderate to high correlations with other relevant measures identified a priori. In particular, the UC-PRO/SS Bowel Signs and Symptoms scale score was strongly correlated with the Partial Mayo Score (*r* = 0.79), IBDQ total score (*r* = 0.70), and IBDQ domain of bowel systems (*r* = 0.73). Both UC-PRO/SS scores also appear to have known-groups validity with significant differences in scores between disease severity groups when defined by the Partial Mayo Score, patient global rating of disease severity, and the clinician global rating of disease severity.

Both scales of the UC-PRO/SS include multiple items to better capture the bowel and abdominal symptom experience of UC from the perspective of the patient, which allows for a more granular assessment of aspects of the disease that are relevant and important to patients. In clinical trials of therapies for UC, the UC-PRO/SS potentially can be used to collect data for a co-primary endpoint or a key secondary endpoint. Therapies targeting inflammation in induction studies could use an objective marker of inflammation (e.g., endoscopy, magnetic resonance enterography, fecal calprotectin) to assess the co-primary or primary endpoint, with the Bowel Signs and Symptoms module as the assessment of a co-primary or key secondary endpoint. Therapies expected to improve functional abdominal symptoms might use this module as the primary endpoint, while maintenance studies of anti-inflammatory studies might use a co-primary endpoint of an objective marker of inflammation and the Bowel Signs and Symptoms and Abdominal Symptoms scales to demonstrate a long-term significant impact on multiple symptom domains important to patients.

Several limitations should be noted for this research. First, although all subjects included in the development and evaluation of the UC-PRO/SS were required to have clinician-confirmed UC based on biopsy, baseline endoscopy was not required for participation in the studies. Thus, it is unclear if subjects were experiencing active inflammation of the colon or rectal mucosa at the time of their participation. Second, the duration of the study was relatively short, limiting information on the variability in UC disease over time, and none of the participants experienced an acute flare up of their condition, thus limiting the data on change, including worsening and improvement. Finally, this was an observational study and not an interventional clinical trial, precluding responsiveness analyses, including tests of sensitivity to change with treatment. Further research is needed to replicate the results presented here in new samples and to determine score sensitivity to change over time with flares and treatment.

## Conclusions

In conclusion, the UC-PRO/SS is a daily diary to gather data on the GI signs and symptoms of UC directly from the patient. The instrument was developed to meet regulatory guidelines, with initial validation evidence suggesting that the UC-PRO/SS scores are reliable, valid, and ready for use and further testing in clinical trials. The UC-PRO/SS complements and extends information provided by the clinician, endoscopy, and biomarkers in clinical studies.

## Additional file

Additional file 1: UC-PRO/SS Supplementary Material. (DOCX 130 kb)
