# Supplementary material for: Development and validation of the Ulcerative Colitis patient-reported outcomes signs and symptoms (UC-pro/SS) diary
Source: J Patient Rep Outcomes. 2018 May 30;2:26. doi: 10.1186/s41687-018-0049-2 (PMC5976680; doi:10.1186/s41687-018-0049-2)
Supplement: Supplementary file 1 — UC-PRO/SS Supplementary Material. (DOCX 130 kb) [file 41687_2018_49_MOESM1_ESM.docx]

**TITLE:** Development and Validation of the Ulcerative Colitis Patient-Reported Outcomes Signs and Symptoms (UC-PRO/SS) Diary

# SupplementARY MATERIAL

## Phase I: Qualitative Research for the Development and Content Validity of the UC-PRO Signs and Symptoms Scale (UC-PRO/SS) and Systemic Symptoms Scale

Initial development of the Ulcerative Colitis Patient-reported Outcomes Signs and Symptoms (UC-PRO/SS) measure and UC-PRO Systemic Symptoms scale were based on qualitative studies conducted by Dr. Peter Higgins at the University of Michigan Health Systems during 2009–2010. The Inflammatory Bowel Disease (IBD) PRO Consortium was formed in early 2011 to develop PRO Drug Development Tools (DDTs) for clinical trials that evaluate the treatment efficacy of IBD, specifically UC and Crohn’s disease. The early work of Dr. Higgins and data from the focus groups were provided to the Consortium. Additional one-to-one concept elicitation interviews and cognitive interviews were performed by Evidera (formerly part of United BioSource Corporation) under the direction of the IBD PRO Consortium. Findings from the focus group discussions and concept elicitation interviews were used to generate items that formed the foundation of the initial UC-PRO/SS and Systemic Symptoms scale.

The development of the UC-PRO/SS measure and Systemic Symptoms scale were based on qualitative studies conducted among a total of 57 patients with UC. To determine instrument content and structure, 42 patients with mild-to-moderately severe UC participated in concept elicitation focus groups (Phase I) or one-to-one qualitative interviews (Phase II). The focus group discussions provided the opportunity to initially explore the important underlying concepts related to symptom experience and the language patients use to describe their condition. Concept elicitation interviews were conducted to obtain in-depth elicitation of symptoms, evaluate saturation of concepts, and obtain additional, expanded information with respect to patient language and variability in symptom experience. Findings from focus group and individual interview-based concept elicitation methods were used to generate an item pool, inform response options, and determine appropriate recall. Two rounds of cognitive interviews were subsequently conducted among 15 patients with UC to further support the saturation and relevancy of items, as well as to refine the measure to ensure clarity and understanding among the target patient population.

All interviews were audio-recorded and transcribed. Content analyses were performed by independent coders, with data organized in NVivo or ATLAS.ti.

### Focus Groups

A total of 33 patients with biopsy-confirmed UC participated in one of six focus groups. Patients were recruited from five clinical sites, located at the University of Michigan, University of North Carolina–Chapel Hill, University of California San Diego, University of Maryland, and Alameda County Medical Center. Patients with UC between the ages of 18 and 75 years with an intact colon were eligible to participate in the study. Patients represented a range of disease activity based on the Simple Clinical Colitis Activity Index (SCCAI) obtained in the prior year, including SCCAI ≤5 (n=3); SCCAI 6–8 (n=5); and SCCAI ≥8 (n=23); SCCAI data were missing for two participants. Experienced scientific staff from the University of Michigan moderated all focus groups using a semi-structured discussion guide to ensure consistency across all focus group discussions. The guide was designed to elicit information about the patients’ UC signs and symptom experiences as they worsened during a flare of their condition.

A total of 19 concepts emerged in the first focus group with additional concepts decreasing rapidly in the subsequent five focus group discussions (number of new concepts emerging totaled 1, 3, 0, 1, and 5 for focus groups 2–6, respectively).

Symptoms reported as worsening with the onset of a flare by more than half of the participants included blood in bowel movement, frequency of bowel movement, pain in the stomach area, consistency of bowel movement (i.e., “liquid watery stools” versus “more solid”), and joint pain/general body pain

Participants in all six focus groups mentioned blood in bowel movement. Participants indicated that the amount of blood in their bowel movement increased and the color of blood became “brighter red” as the severity of their flare-up worsened. Participants described blood in bowel movement as being a “game changer” and the signal that the condition was serious and required attention. Participants in all six focus groups mentioned frequency of bowel movements as an important symptom. The frequency of bowel movements reported included a range of responses such as “every hour,” “20–40 times a day,” “5–20 times a day,” to “3–5 times a day” depending on the participant, course of treatment, and severity of flare.

In every focus group, participants mentioned experiencing pain, predominately in the stomach area and joints. Pain in the stomach area was described as “cramping pain,” “stomach pain,” “abdominal pain,” pain in the “lower abdomen,” and “on the sides.” The type and onset of pain in the stomach area varied by individual, but the common themes among patients were intensity of the pain, feeling worse after eating, and having to use the bathroom. Other bodily pain was primarily limited to the joints, with patients using general expressions such as “joints” and “joint pain,” as well as joint pain specific to the “hands,” “knees,” and “feet.” The pain expressions were varied and included “extreme,” “excruciating,” “dull and constant,” “stabbing,” and “constant pressure.”

Participants in every focus group also mentioned stool consistency as an important symptom related to their UC. Participants described stool consistency as being, “loose,” “diarrhea,” “watery,” and “soft.” Stool consistency was mentioned in conjunction with experiencing a flare, but was also a common and normal occurrence when other UC symptoms improved. Feeling tired also was mentioned in every focus group as being a symptom associated with UC. Patient descriptors included “worn out,” “tired,” “fatigue,” “exhausted,” and “napping all day.” Other important symptoms included urge/need to have a bowel movement right away, mucus in bowel movement, gas, leakage/accidents, weakness, bloating, dehydration, and lack of appetite

### Qualitative Interviews

While focus groups allow for a dynamic interchange between participants, which often leads to a broader range of information discussed, patient language and opinions may become influenced by other group members. Individual interviews, on the other hand, provide an opportunity to explore, in more depth, concepts elicited during the focus group exchange, as well as an opportunity to more accurately track important underlying concepts obtained spontaneously by individuals. Thus, to ensure saturation on important signs and symptoms of UC from the perspective of the patient and extend results of Phase I, nine one-to-one qualitative interviews were conducted with adults with biopsy-confirmed UC, recruited from two GI clinics, one in Poughkeepsie, NY, the other in Torrance, CA. Participants had been diagnosed with UC for at least six months prior to screening and included five participants with a Partial Mayo Score 2–4 and four with a Partial Mayo Score ≥4; those with an external fistula, ileostomy, colostomy, or intra-abdominal surgery in the previous four months were excluded.

A semi-standardized interview guide was used by the interviewer to ensure consistency across all subjects. The purpose of the interviews was three-fold: 1) identify important UC symptoms obtained spontaneously from patients using open-ended probes; 2) further explore the frequency and variability of symptom experience as identified by the patient; and 3) inform the development of response options and appropriate recall of the symptom measure. Discussion focused on participants’ current experiences, their experiences during an episode or flare-up, and the impact of these symptoms on their daily life.

Similar to findings from the focus groups, important symptoms included frequency of bowel movements, blood in bowel movements, consistency of bowel movements, mucus in bowel movement, pain in stomach area, joint pain/general body pain, tiredness, urge/need to have a bowel movement right away, gas, leakage/accidents, bloating, dehydration, and lack of appetite. The symptoms that were most relevant during episodes of flare included blood in bowel movements, frequency of bowel movements, consistency of bowel movements, general body pain/joint pain, and urge/need to have a bowel movement right away, also consistent with findings from the focus group discussions.

Patient descriptions of the symptoms they experienced during a flare were similar to language that they used to describe their everyday symptoms, just more severe and/or persistent. In addition, patients noted that the severity of a symptom could vary during a flare episode within the day (particularly with respect to pain, urge/need to have a bowel movement right away, and bloating), or day to day. Patient descriptions of their symptom experience, including duration of an acute attack, underline the variability not only within, but also between patients.

Findings from the focus group discussions and one-to-one qualitative interviews indicate that data were gathered to the point of saturation (Figure S1), with comprehensive results obtained by using the two elicitation methods.

Figure S1. Saturation of Concepts

Abbreviations: BM = bowel movement

### Development of Rating Scale

Based on findings from the focus group discussions, qualitative interviews, and input from clinical experts, a list of relevant items was generated, forming the foundation of the draft of UC-PRO/SS measure and Systemic Symptoms scale. Instructions, item stems, and response options were derived from patient language elicited during focus groups and interviews to ensure appropriateness, relevance, understanding, and clarity of the items included in the measure. A list of the symptom items and rationale for item wording based on focus group discussion, concept elicitation interviews, and cognitive interviews is included in Table S1.

Table S1. Symptom Items and Rationale for Wording

| Symptom Items | Rationale for Wording |
| --- | --- |
| Number of bowel movements | - Patient language - Use “bowel movement” instead of “stool” to describe number of times going to the toilet (and including “loose, watery stools” and “diarrhea”) |
| Bowel movements mostly or completely liquid (frequency) | - Based on patient descriptions of “diarrhea,” “runs,” “loose watery stools,” and other similar expressions |
| Blood in your bowel movements (frequency) | - Patient language |
| Mucus (white material) in your bowel movements (frequency) | - Patient language |
| Stool, blood, or liquid leaked out before you reached a toilet (frequency) | - Based on patient descriptions of “accidents,” “not reaching the toilet in time,” and other similar expressions |
| Passing gas (frequency) | - Patient language |
| Feel the need to have a bowel movement right away (frequency) | - Based on patient descriptions of “urgency,” “urge to go right away,” and other similar expressions |
| Pain in your belly (severity) | - Capture pain in stomach area - Belly was consistent with patient language to describe pain in stomach area and tested in cognitive interviews for comprehension |
| Feel bloating in your belly (severity) | - Patient language “bloating” - Belly was consistent with patient language to describe pain in stomach area and tested in cognitive interviews for comprehension |
| Feel pain in your knees, hips, and/or elbows (severity) | - Based on patient descriptions of general body pain and joint pain (not specific to stomach area) - Excludes joints in fingers/hands associated with arthritic pain |
| Feel tired (severity) | - Patient language |
| Lack an appetite (severity) | - Patient language |
| Feel weak (severity) | - Patient language - Use “BM” instead of “stool” to describe number of times going to the toilet (and including “loose, watery stools” and “diarrhea”) |
| Feel thirsty (severity) | - Based on patient descriptions of “diarrhea,” “runs,” “loose watery stools,” and other similar expressions |

Data from the psychometric evaluation study were used to inform scoring and evaluate the psychometric properties of UC-PRO/SS domains (Bowel Signs and Symptoms and Abdominal Symptoms) and Systemic Symptoms scale.

### Cognitive Interviews and Modifications

Two rounds of one-to-one interviews were conducted with UC patients (N=15), using cognitive interview methodology, examining readability and comprehensiveness. In each case, the cognitive interview followed the participant’s completion of the questionnaire and used a standardized discussion guide. For both rounds of cognitive interviews, subjects were first asked if there were other symptoms they experienced that were not captured on the questionnaire to confirm concept saturation. Subjects were then cognitively interviewed to establish: 1) the clarity of the items; 2) how the respondents interpret the items and how well their understanding matches the concept purportedly measured by the item; 3) ease of completion of the items; 4) the comprehensiveness of the instrument; and 5) the appropriateness of the format, response scales, and recall period. Eligibility criteria were the same as that used in the one-to-one concept elicitation qualitative interviews. All interviews were audio-recorded and transcribed. Content analyses were performed by independent coders, with data organized in ATLAS.ti.

The first round of cognitive interviews included ten patients with UC (confirmed diagnosis based on biopsy) recruited through three clinical sites specializing in gastroenterology, located in Poughkeepsie, NY; Torrance, CA; and Charlotte, NC. No new concepts emerged during the ten interviews. All patients indicated that the UC-PRO/SS and Systemic Symptoms scales captured all the relevant symptoms they experienced. Overall, participants reported that the pen-paper version of the scales were easy to complete and understand, with items relevant to the symptoms that they experience. The instructions, item content, and response scales were clear and well-understood, with a few exceptions. For the symptoms blood in bowel movement; mucus in bowel movement; stool, blood, or liquid leaking; and passing gas, it was unclear how patients were interpreting the response options related to symptom severity. With respect to recall, patients felt that daily recall period (past 24 hours) was appropriate and realistic for rating the presence and severity of this and other UC symptoms. Based on findings, the following modifications were made:

- Blood in bowel movement; mucus in bowel movement; stool, blood, or liquid leaking: Response options revised from severity to frequency (yes/no, rarely, sometimes, often, always) to better reflect patient experience; instructions modified accordingly
- Passing gas: Response options revised from severity to frequency (yes/no, rarely, sometimes, often, very often) to better reflect patient experience; instructions modified accordingly
- Bowel movements mostly or completely liquid: Response option “not at all” revised to “never.” Although subjects did not indicate a problem with this response category, “not at all” reflects severity and is inconsistent with other response categories that address frequency (yes/no, rarely, sometime, often, always)
- For symptom items using severity response options, “very mild” was deleted, as it was felt that it would be difficult for patients to distinguish between “very mild” and “mild” symptoms
- Pain in belly; bloating in belly; need to have bowel movement right away; pain in knees, hips, and/or elbows: To address variability within a 24-hour period, instructions modified from “If Yes, how severe was this symptom” to “If Yes, how severe was this symptom at its worst”

The second round of five additional cognitive interviews was conducted to confirm changes and to further explore patient understanding and relevancy of the items and corresponding response options. Round 2 also provided an opportunity to examine patient understanding of the scales formatted as ePRO screen shots, with one item per page. No new concepts emerged during the second round of cognitive interviews, with patients confirming that the UC-PRO/SS and Systemic Symptoms scale captured all the relevant symptoms they experienced. Patients found the revised scales and ePRO format easy to complete and understand. Findings indicate that patients understood the items as intended. The recall period and response options were found to be appropriate. Based on findings from Round 2, no additional modifications were made.

The preliminary conceptual framework, based on qualitative data and clinical expert input, outlines a score for UC Signs and UC Symptoms, with the possibility of a combined score of UC signs and symptoms (Figure S2).

Figure S2. Preliminary Hypothesized Conceptual Framework Based on Qualitative Research

## Abbreviations: BM = bowel movement

Frequency of BMs mostly or completely liquid

Number of BMs

Frequency of blood in BM

UC

Signs

Frequency of stool, blood, or liquid leaking

Frequency of passing gas

Severity of need to have a BM right away

Severity of pain in belly

Severity of bloating in belly

UC

Symptoms

Severity of pain in knees, hips, and/or elbows

Severity of feeling tired

Severity of lack of appetite

Severity of feeling weak

Severity of feeling thirsty

Frequency of mucus in BM

Systemic Symptoms

UC

Signs and Symptoms

**General Concepts**

**Item**

**-**

**Level Concept**

Systemic Symptoms

## Phase II: Quantitative Research

Supplemental results from the quantitative analysis for the UC-PRO/SS Measure and Systemic Symptoms Scale are provided in Tables S2–S9 and Figures S3 and S4.

Table S2. Item Analysis: Systemic Symptom Item Descriptive Characteristics at Worst Day between Visit 1 and Visit 2 (N=198)

| **Systemic Symptom Items** | **N** | **Mean (SD)** | **Range** | **Floor (%)** | **Ceiling (%)** | **% Missing** |
| --- | --- | --- | --- | --- | --- | --- |
| Pain in knees, hips, elbows | 198 | 1.0 (1.18) | 0–4 | 103 (52.0%) | 6 (3.0%) | 0 (0.0%) |
| Feel tired | 198 | 1.6 (1.27) | 0–4 | 58 (29.3%) | 15 (7.6%) | 0 (0.0%) |
| Lack appetite | 198 | 0.8 (1.14) | 0–4 | 126 (63.6%) | 5 (2.5%) | 0 (0.0%) |
| Feel weak | 198 | 1.1 (1.23) | 0–4 | 99 (50.0%) | 7 (3.5%) | 0 (0.0%) |
| Feel thirsty | 198 | 1.4 (1.24) | 0–4 | 66 (33.3%) | 11 (5.6%) | 0 (0.0%) |

Table S3. Exploratory Factor Analysis Standardized Factor Loadings

| **Item** | **Factor 1** | **Factor 2** | **Factor 3** |
| --- | --- | --- | --- |
| Number of bowel movements | **0.866** | -0.028 | -0.049 |
| Mostly liquid bowel movements | **0.789** | -0.036 | 0.066 |
| Blood in bowel movements | **0.560** | 0.131 | 0.061 |
| Mucus in bowel movements | ***0.352*** | *0.370* | 0.004 |
| Leak before reach toilet | **0.794** | 0.017 | -0.151 |
| Need to have bowel movements | ***0.468*** | 0.036 | *0.251* |
| Pass gas | -0.107 | 0.439 | **0.168** |
| Pain in belly | 0.010 | 0.003 | **1.072** |
| Bloating in belly | 0.081 | *0.359* | ***0.326*** |
| Pain in knee, hips, elbows | -0.105 | **0.777** | -0.012 |
| Feel tired | -0.180 | **0.920** | 0.002 |
| Lack appetite | -0.034 | **0.801** | 0.058 |
| Feel weak | 0.026 | **0.997** | -0.159 |
| Feel thirsty | 0.045 | **0.548** | 0.017 |

NOTE: Items in bold were retained in final factor solution. Items in italics met criteria for cross-loading (there is less than 50% difference between the largest and 2d-largest factor loadings).

Comparative Fit Index (CFI) = 0.975, Root Mean Square of Approximation (RMSEA) = 0.07, and Standardized Root Mean Square Residual = 0.046

Systemic Symptoms Scale goodness of fit statistics: CFI= 1.00; RMSEA = 0.00; and Weighted Root Mean Residual = 0.253

Table S4. Internal Consistency Reliability: Visit 2

| **Scales** | **N** | **Cronbach’s α** | **Cronbach’s α  If Item Deleted** |
| --- | --- | --- | --- |
| **Bowel Signs and Symptoms** | 181 | 0.80 |  |
| Number of bowel movements |  |  | 0.73 |
| Bowel movements mostly or completely liquid |  |  | 0.73 |
| Blood in bowel movements |  |  | 0.78 |
| Mucus in bowel movements |  |  | 0.80 |
| Stool, blood, or liquid leakage |  |  | 0.77 |
| Feel need to have bowel movements right away |  |  | 0.78 |
| **Abdominal Symptoms** | 181 | 0.66 |  |
| Pass gas |  |  | 0.79 |
| Pain in belly |  |  | 0.42 |
| Bloating in belly |  |  | 0.42 |
| **Systemic Symptoms** | 181 | 0.79 |  |
| Pain in knees, hips, and/or elbows |  |  | 0.76 |
| Feel tired |  |  | 0.71 |
| Lack of appetite |  |  | 0.76 |
| Feel weak |  |  | 0.70 |
| Feel thirsty |  |  | 0.78 |

Abbreviations: α = alpha; N = number

Table S5. Test-Retest Reliability among Stable Subjects^1^

| **UC-PRO/SS** | **N** | **Visit 2**  **Mean (SD)** | **Visit 3**  **Mean (SD)** | **Difference^2^** | **P value from paired T test** | **ICC** |
| --- | --- | --- | --- | --- | --- | --- |
| Bowel Signs & Symptoms | 77 | 1.32 (0.89) | 1.26 (0.84) | -0.06 (0.54) | 0.3600 | 0.81 |
| Abdominal Symptoms | 77 | 1.40 (0.71) | 1.36 (0.73) | -0.04 (0.53) | 0.5369 | 0.74 |
| Systemic Symptoms | 77 | 0.89 (0.65) | 0.81 (0.62) | -0.08 (0.48) | 0.1445 | 0.71 |

^1^ Stable subjects based on PGRC

^2^ Mean Difference = Visit 3–Visit 2 (using seven-day average score prior to Visits).

Abbreviations: ICC= intraclass correlation coefficient; N = number; SD = standard deviation; UC-PRO/SS = Ulcerative Colitis Patient-reported Outcome Signs and Symptoms

Table S6. Correlations between Systemic Symptom Score and Other Clinical Variables^1,2^

| Clinical Variable | Systemic Symptoms r (p) |
| --- | --- |
| *Clinician Ratings:* |  |
| Partial Mayo Score | 0.41 (<0.0001) |
| Clinician Global Rating of Disease Severity | 0.41 (<0.0001) |
| *Patient Ratings:* |  |
| Patient Global Rating of Disease Severity | 0.55 (<0.0001) |
| IBDQ |  |
| Total score | -0.62 (<0.0001) |
| Bowel systems | -0.55 (<0.0001) |
| Emotional health | -0.58 (<0.0001) |
| Systemic systems | -0.64 (<0.0001) |
| Social function | -0.54 (<0.0001) |
| WPAI-SHP |  |
| Absenteeism | 0.30 (0.0004) |
| Presenteeism | 0.46 (<0.0001) |
| Work productivity loss | 0.53 (<0.0001) |
| Activity impairment | 0.52 (<0.0001) |
| PROMIS |  |
| Global physical health | -0.46 (<0.0001) |
| Global mental health | -0.46 (<0.0001) |
| General health | -0.45 (<0.0001) |
| Satisfaction with social role | -0.40 (<0.0001) |
| BPI – Worst Pain | 0.60 (<0.0001) |

^1^ Spearman’s correlation coefficients.

^2^ Seven-day average scores used for Systemic Symptoms scale used. The Systemic Symptoms scale is scored as a simple mean across all items comprising the scale. Weekly scores are computed by taking the mean across 7 days, with at least four days of complete data required for computation. If more than 4 days of data are missing, the weekly average score is set to missing.

Abbreviations: BPI = brief pain inventory; IBDQ = Inflammatory Bowel Disease Questionnaire; p = P value; PROMIS = Patient Reported Outcomes Measurement Information System; r = rating; WPAI-SHP = Work Productivity and Activity Impairment—Specific Health Problems

Table S7. Known-groups Validity: UC-PRO/SS and Systemic Symptom Scores by Partial Mayo Score; Visit 2^1^

|  | Three-item Partial Mayo Score | | | Overall *F* Value^2^ (P value) | Pairwise Comparisons^3^ |
| --- | --- | --- | --- | --- | --- |
|  | **Mild**  **Score 1–2** | **Moderate**  **Score 3–5** | **Severe**  **Score ≥6** |  |  |
|  | **N**  **Mean (SD)** | **N**  **Mean (SD)** | **N**  **Mean (SD)** |  |  |
| Bowel Signs and Symptoms | 52  0.6 (0.4) | 84  1.2 (0.6) | 52  2.2 (0.7) | 50.05 (<0.0001) | 1**** 2**** 3**** |
| Abdominal Symptoms | 52  1.1 (0.5) | 84  1.5 (0.7) | 52  1.8 (0.8) | 8.09 (<0.0001) | 1*** 2**** 3* |
| Systemic Symptoms | 52  0.6 (0.5) | 84  0.9 (0.6) | 52  1.3 (0.8) | 8.35 (<0.0001) | 1** 2**** 3* |

^1^ Seven-day average score.

^2^ An analysis of covariance (ANCOVA) model adjusting age, and sex.

^3^ p values are: *<0.05, **<0.01, ***<0.001, **** <0.0001. Comparisons are 1= Mild vs. Moderate, 2= Mild vs. Severe, 3= Moderate vs. Severe.

Abbreviations: N = number; SD = standard deviation; UC-PRO/SS = Ulcerative Colitis Patient-reported Outcome Signs and Symptoms

Table S8. Known-groups Validity: UC-PRO/SS and Systemic Symptom Scores by Patient Global Rating of Disease Severity; Visit 2^1^

|  | Patient Global Rating of Disease Severity | | | | Overall *F* Value^2^  (P value) |
| --- | --- | --- | --- | --- | --- |
|  | **< Median** | | **≥ Median** | |  |
|  | **N** | **Mean (SD)** | **N** | **Mean (SD)** |  |
| Bowel Signs and Symptoms | 93 | 0.9 (0.6) | 95 | 1.7 (0.8) | 23.51 (<0.0001) |
| Abdominal Symptoms | 93 | 1.2 (0.6) | 95 | 1.8 (0.8) | 10.08 (<0.0001) |
| Systemic Symptoms | 93 | 0.7 (0.5) | 95 | 1.2 (0.7) | 13.76 (0.0001) |

^1^ Seven-day average score.

^2^ An analysis of covariance (ANCOVA) model adjusting age and sex.

Abbreviations: N = number; SD = standard deviations; UC-PRO/SS = Ulcerative Colitis Patient-reported Outcome Signs and Symptoms

Table S9. Known-groups Validity: UC-PRO/SS and Systemic Symptom Scores by Clinician Global Rating of Disease Severity; Visit 2^1^

|  | Clinician Global Rating of Disease Severity | | | | Overall *F* Value^2^  (P value) |
| --- | --- | --- | --- | --- | --- |
|  | **< Median** | | **≥ Median** | |  |
|  | **N** | **Mean (SD)** | **N** | **Mean (SD)** |  |
| Bowel Signs and Symptoms | 61 | 0.7 (0.5) | 127 | 1.6 (0.8) | 21.51 (<0.0001) |
| Abdominal Symptoms | 61 | 1.1 (0.7) | 127 | 1.7 (0.7) | 8.26 (<0.0001) |
| Systemic Symptoms | 61 | 0.6 (0.5) | 127 | 1.1 (0.7) | 9.36 (<0.0001) |

^1^ Seven-day average score.

^2^ An analysis of covariance (ANCOVA) model adjusting age and sex.

Abbreviations: N = number; SD = standard deviation; UC-PRO/SS = Ulcerative Colitis Patient-reported Outcome Signs and Symptoms

Figure S3. Final Conceptual Framework for the UC-PRO/SS

Number of bowel movements

Frequency of bowel movements mostly or completely liquid

Frequency of blood in bowel movement

Frequency of mucus in bowel movement

Severity of need to have bowel movements right away

Frequency of stool, blood, or liquid leaking

Bowel Signs and Symptoms Scale

Frequency of passing gas

Severity of pain in belly

Severity of bloating in belly

Abdominal Symptoms Scale

**General Concepts**

**Item-Level Concept**

Figure S4. Final Conceptual Framework for the Systemic Symptoms Scale

Severity of pain in knees, hips, and/or elbows

Severity of feeling tired

Severity of lack of appetite

Severity of feeling weak

Severity of feeling thirsty

Systemic Symptoms Scale

**General Concepts**

**Item-Level Concept**
